# Effect and mechanism of acupuncture on Alzheimer’s disease: A review

**DOI:** 10.3389/fnagi.2023.1035376

**Published:** 2023-03-03

**Authors:** Liu Wu, Yuting Dong, Chengcheng Zhu, Yong Chen

**Affiliations:** ^1^Department of Tuina, Hospital of Chengdu University of Traditional Chinese Medicine, Chengdu, China; ^2^School of Acupuncture and Tuina, Chengdu University of Traditional Chinese Medicine, Chengdu, China; ^3^Department of Galactophore, Hospital of Chengdu University of Traditional Chinese Medicine, Chengdu, China; ^4^Department of Emergency, Hospital of Chengdu University of Traditional Chinese Medicine, Chengdu, China

**Keywords:** Alzheimer’s disease, senile dementia, mechanism of action, acupuncture therapy, curative effect

## Abstract

With the development trend of an aging society, Alzheimer’s disease (AD) has become an urgent problem in the field of medicine worldwide. Cognitive impairment in AD patients leads to a decline in the ability to perform daily living and abnormalities in behavior and personality, causing abnormal psychiatric symptoms, which seriously affect the daily life of patients. Currently, mainly drug therapy is used for AD patients in the clinic, but a large proportion of patients will experience drug efficacy not working, and even some drugs bring severe sleep disorders. Acupuncture, with its unique concept and treatment method, has been validated through a large number of experiments and proved its reliability of acupuncture in the treatment of AD. Many advances have been made in the study of the neurobiological mechanisms of acupuncture in the treatment of AD, further demonstrating the good efficacy and unique advantages of acupuncture in the treatment of AD. This review first summarizes the pathogenesis of AD and then illustrates the research progress of acupuncture in the treatment of AD, which includes the effect of acupuncture on the changes of biochemical indicators in AD *in vivo* and the specific mechanism of action to exert the therapeutic effect. Changes in relevant indicators of AD similarly further validate the effectiveness of acupuncture treatment. The clinical and mechanistic studies of acupuncture in the treatment of AD are intensified to fit the need for social development. It is believed that acupuncture will achieve new achievements in the treatment of AD as research progresses.

## Introduction

Alzheimer’s disease (AD) is a chronic brain degenerative disease characterized by progressive distant and near memory impairment, decreased ability of analysis and judgment, emotional changes, behavioral disorders, and even disturbance of consciousness. it is one of the major diseases seriously endangering the health of the elderly (Pfundstein et al., 2022; Rodini et al., 2022). The etiology of AD is related to many factors. The typical pathological changes are a large number of senile plaques (SP) between nerve cells, neurofibrillary tangles (NFTs) in nerve cells, and neuronal loss. Among all dementia patients, AD patients are the most common and account for the vast majority (about 60–70% of the total number of dementia). Autopsy studies of dementia show that 50 of 70% of the patients are AD (Aborode et al., 2022; Flores et al., 2022). Recent reports have shown that there are about 46 million AD patients worldwide, which will double every 20 years in the future. AD poses a serious threat to the health and safety of the elderly. Thirty years later, it is estimated that the global number of AD patients will reach an alarming 131.5 million. The incidence of AD was positively correlated with age, and there were more females than males. Since the 1980s, the epidemiological survey data on the prevalence of AD in various countries around the world are relatively similar, and the prevalence of dementia in people over 65 years old is about 5% (Eid et al., 2022; Kishino et al., 2022). AD is present at a higher prevalence of 20% in individuals older than 80 years, and the prevalence increases with age. The highest prevalence of AD in people aged 60 and over in Europe and North America was 5.4 and 6.4%, respectively; 4.9% in Latin America; 3.8 and 3.9% in Eastern Europe; China 4%.

At present, for this intractable type of disease, there are no effective drugs and technical means for clinical treatment given at home and abroad (Martersteck et al., 2022; Ruengchaijatuporn et al., 2022). There are currently several claims in the clinic to the related pathogenesis of Alzheimer’s disease, namely the cholinergic doctrine, the tau protein hypothesis, the neurovascular doctrine, the oxidative stress doctrine, the β- Amyloid theory, the brain-gut axis theory, etc., whether brain extracellular amyloid peptide exists β (Aβ) Deposition and intracellular tau protein (Tau) hyperphosphorylation while neurofibrillary tangles are the pathological diagnostic criteria of the disease, but the exact etiology of the AD is not well understood, and an effective cure for the AD is lacking to date. AD is a multi-factor and multi-mechanism disease, which has posed a serious threat to human health. As an important part of traditional medicine, acupuncture has the characteristics of multi-target, multi-way, and multi-level functions. Similarly, there are many clinical studies on acupuncture for the treatment of AD. Li R. et al. (2020) randomized 60 AD patients into treatment and control groups, 30 each. Patients in both groups were treated with oral donepezil hydrochloride and conventional therapy. The control group was given conventional acupuncture. In the treatment group, “Fengchi,” “Tianzhu,” “Wangu,” “Panfeng,” “Fengfu,” and “Zhongwan” acupoints were sampled. In the control group, they were treated with common acupuncture, including “Baihui,” “Sishencong,” “Intang,” “Shenting,” “Taixi,” and “Xuanzhong” acupoints. The overall response rate was 82.1% in the treatment group and 72.4% in the control group. Moreover, cognition and memory were significantly improved after treatment.

This article reviews the recent studies on acupuncture and moxibustion in the treatment of AD at home and abroad. It is found that acupuncture can regulate the overall regulation of AD by regulating abnormal protein expression in the brain, regulating the physiological and pathological state of microglia, regulating mitochondrial autophagy, regulating epigenetic modification, giving full play to neuron protection, improving synaptic plasticity, regulating oxidative stress and regulating energy metabolism. In-depth analysis of the effect mechanism of acupuncture and moxibustion in the treatment of AD, and reveal its deep action principle, to provide a scientific and reasonable theoretical basis for clinical diagnosis and treatment of AD.

## Research status of action mechanism

### Pathways and effects of acupuncture in the treatment of AD

From the perspective of traditional Chinese medicine, neurodegenerative diseases are diseases of the brain. At present, numerous studies have shown that acupuncture at the “Baihui” acupoint can promote the treatment of encephalopathy. The acupoint selection criteria for the treatment of memory disorders, except the “Baihui” acupoint, are based on the symptoms of the brain disease itself and associated underlying diseases (de Pins et al., 2019; Li G. et al., 2019).

Acupuncture stimulation intensity and frequency are also important for the treatment of diseases. Found that high-intensity acupuncture at “Baihui,” and “Dazhui” acupoints improved the learning and memory function of AD rats better than low-intensity acupuncture. Wang et al. (2020c) studied the therapeutic effects of acupuncture “Baihui” and “Shenshu” at different frequencies (50, 30, and 2 Hz) on AD rats and the underlying mechanisms. The results showed that acupuncture downregulated GSK-3β levels in the hippocampus of AD Rats, upregulated GAP-43 levels, and 50 Hz acupuncture improved learning and memory function and repaired synaptic damage in AD rats better than 30 and 2 Hz (Lin et al., 2018).

Combination drugs have also tried to improve the effect of acupuncture on memory impairment. Yang et al. (2021) explored the efficacy and mechanism of a combination of electroacupuncture at “Baihui,” and “Yintang” acupoints and donepezil in the treatment of AD. The results showed that acupuncture enhanced the effect of donepezil on improving learning and memory function in AD rats and facilitated the transport of donepezil through the blood-brain barrier by regulating the expression of matrix metalloproteinase nine, low-density lipoprotein receptor-related protein one and PGP Aβ (Wegmann et al., 2018).

Acupuncture at the acupoints “Baihui,” “Dazhui,” and “Zusanli” combined with gastrodin in the treatment of learning and memory impairment in AD rats. Acupuncture or gastrodin both improved cognitive function and upregulated SIRT1, Bcl2, and PGC-1α in AD rats expression, inhibiting the expression of Bax, and protecting hippocampal neurons, but the effect of combined acupuncture and gastrodin was better than that of acupuncture or gastrodin alone. Interestingly, laser acupuncture improved cognitive impairment induced by cerebral ischemia and modulated the expression of CREB, BDNF, Bcl2, and Bax genes, exerting neuroprotective effects. In addition, some physical therapies, such as transcranial magnetic stimulation, moxibustion, massage, and rehabilitation therapy, can also improve the effect of acupuncture, which needs attention (Jiang et al., 2017; Guo et al., 2020; Hang et al., 2022).

**FIGURE 1:**
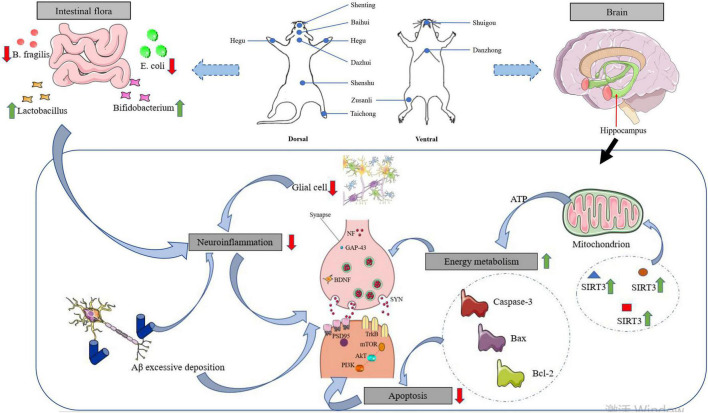
Effects of acupuncture intervention on neuroplasticity in AD pathogenesis. With the focus on synaptic plasticity in the hippocampus, the mechanisms by which acupuncture affects memory impairment are reviewed in terms of synaptic proteins, ad signature proteins, gut microflora, neuroinflammation, and energy metabolism.

As shown in Figure 1, the mechanism of action of acupuncture is closely related to the repair of synaptic plasticity in the hippocampus. This review focuses on the pathogenesis of AD (synaptic proteins, ad signature proteins, gut flora, neuroinflammation, neuronal apoptosis, and changes in energy metabolism) and the role of acupuncture in the treatment of AD. It is also worth highlighting that synaptic plasticity in the hippocampus may be a central and common link to the above mechanisms.

### Acupuncture intervention modulates central neurotransmitter release

Neurobiochemistry reveals that amino acid neurotransmitters are hardly related to learning and memory (Al-Nasser et al., 2022). Glutamate (Glu) is the capital excitatory neurotransmitter of pyramidal neurons, which acts an essential role in learning and memory, synaptic plasticity in development, neuronal survival, and dendritic growth and degeneration. Its function is regulated by N-methyl-D-aspartate (NMDA) receptor. Long-term synaptic enhancement (LTP) is considered to be a physiological mechanism of information storage and memory formation in the brain (Khavinson et al., 2021). DMNA receptors are considered to be closely related to learning and memory due to the existence of LTP. The relationship between Glu and LTP can be summarized as follows: stimulation-> postsynaptic NMDA receptor activation-> channel opening-> Ca^2+^ influx-> cell membrane depolarization-> LTP (Catarzi et al., 2007). There are excitatory amino acids and inhibitory amino acids in the brain, which play the role of neurotransmitters and act a crucial role in the process of learning and memory (Noda, 2016; Du et al., 2020).

The pathogenesis of AD associated with changes in excitatory amino acid content in brain tissue has long been demonstrated. Tang et al. (2005) used alginic acid injection to make a rat dementia model, acupuncture “Dazhui,” “Shenshu,” “Taixi,” and “Housanli” acupoints to observe the function of acupuncture on the content of Glu and aspartic acid (Asp) in the brain of senile dementia rats. It was detected by high-performance liquid chromatography and ultraviolet spectrophotometer colorimetry. The results showed that the level of Glu and Asp in the brain tissue of the model group were significantly lower than those of the sham operation group (*P* < 0.01), suggesting that the changes in Glu and Asp contents were closely related to the pathogenesis of AD, which was consistent with the related experiment outcome (Hynd et al., 2004; Natale et al., 2006). After acupuncture and dihydroergotamine methanesulfonamide (Hydergine) treatment, the level of Glu and ASP in the brain of model rats decreased significantly (*P* < 0.05), suggesting that increasing the content of excitatory amino acids in the brain of senile dementia rats may be one of the effective mechanisms of acupuncture in the treatment of senile dementia. According to AD’s glutamatergic hypothesis, the increase of synthesis and release of excitatory amino acids (EAAS), especially Glu, leads to excitatory neurotoxicity, which leads to neuronal degeneration and death is an important mechanism of AD brain degeneration (O’Neill et al., 2004). Shi and Zhuang (2020) observed the synthesis and release of EAAS in different brain regions by acupuncture at “Shuigou” and “Neiguan” acupoints of SAM-P/8 mice, the results point out that acupuncture could decrease the abnormally increased content of Glu, Asp, and glutamine, suggesting that the regulating effect of acupuncture on EAAS metabolism may be one of the important mechanisms in the treatment of AD.

### Acupuncture intervention modulates the activity of acetylcholine (ACh), acetylcholine transferase (ChAT), and acetylcholinesterase (AChE)

It has been found that the cholinergic projection system from the basal forebrain nucleus to the cerebral cortex and hippocampus is called the forebrain cholinergic system, which is closely related to advanced neural activities such as learning and memory (Zhang et al., 2022). The most obvious changes in the central cholinergic system of AD are the basal forebrain cholinergic system, including the basal nucleus of Meynert and the medial septal nucleus, which project to an extensive region of the hippocampus and cerebral cortex. About 90% of cholinergic neurons are lost in the basal forebrain of patients with AD (Li, 2022). Neurobiochemical studies have shown that Ach synthesis and ChaT activity are decreased in patients with AD, which is inextricably linked to the progression of dementia.

The degeneration of the cholinergic system in the basal forebrain may be the major cause of cognitive impairment in patients with AD (Gu and Wang, 2021; Letsinger et al., 2022). Wang and Zhou (2009) established the AD model by microinjection of Aβ1-40 into bilateral Meynert basal nuclei of rats to study the effect of acupuncture on the contents of Ach, ChAT, and AchE in the brain of AD model rats. After acupuncture at “Baihui,” “Zusanli,” and “Shenshu” acupoints for 1 month, to test the function of acupuncture on the synthesis and decomposition of ACh in the hippocampus and cortex, it is important to detect the activity and content of ache, ACh, and chat It was found that the activity of ChAT and the content of Ach in the cortex and hippocampus of AD rats induced by Aβ1-40 decreased significantly, while the activity of AchE increased significantly, and the rate of AchE and Ach in brain tissue increased significantly, which was consistent with AD’s cholinergic theory (Vecchio et al., 2021). The outcome points out that the level of activities of Ach, ChAT, and AchE in the cortex and hippocampus in the acupuncture group were different from those in the model group (*P* < 0.01). It has been proved that acupuncture can increase the activity of ChAT, inhibits the activity of AchE, promote the synthesis of Ach, inhibit the decomposition of Ach, increases the content of Ach in brain tissue, and reverse memory loss (Reale and Costantini, 2021). Yu et al. (2021) used scopolamine injection to make a senile dementia model in rats and mice, and acupuncture at the “Yongquan” acupoint was carried out alternately for 30 days. The activity of AchE in the brain of senile dementia mice and the hippocampus of senile dementia rats were detected, respectively. The experimental demonstration that the activity of AchE in the brain of mice in the scopolamine model group was significantly lower than that in the control group (*P* < 0.05), and the brain AchE in the model group was significantly higher than that in the model group after acupuncture (*P* < 0.05). The hippocampal AchE of senile dementia model rats was significantly lower than that of the control group, and acupuncture had a significant effect on hippocampal AchE response of senile dementia model rats, which was significantly different from that of the model group. In a word, it is suggested that acupuncture has a significant function on the activity of acetylcholinesterase in the brain of senile dementia mice and the hippocampus of senile dementia rats (Sutalangka et al., 2013; Yang Q. et al., 2019; Zhang et al., 2019).

### Acupuncture modulates monoamine neurotransmitter release

Monoamine neurotransmitters in brain tissue include: 5-HT, NA, and DA, 5-HT are important neurotransmitters for maintaining normal intelligence. The brain system of patients with AD was severely damaged, with an average reduction of 61% of the concentration of 5-HT in the frontal lobe (Plini et al., 2021; Tripathi and Mazumder, 2021). NA is widely projected to the entire central nervous system through axonal connections, participates in the regulation of the excited state of the entire cerebral cortex, and has a wide range of effects on arousal, sensory, emotional, and higher cognitive functions.

The activity of NA in the cerebral cortex of AD decreased significantly, and the loss of NA neurons and the change in NA activity were related to the severity of AD. Ma et al. (2020) made an AD model by microinjection of 6-hydroxydopamine into the ascending dorsal tract of NA in the rat brain, resulting in the decrease of learning and memory function in rats, which proved that the levels of central NA and DA were closely related to learning and memory. Bao and Lv (2003) used Alcl3 to replicate the senile dementia model of chronic aluminum poisoning, acupuncture at “Fengfu” acupoint for 14 days, and drug control treatment with nimodipine tablet (NM), intragastric administration once a day for 14 days (Kaur et al., 2021). The contents of monoamine neurotransmitters 5-HT, NA, and DA in brain tissue were determined, and the behavior test was carried out by a Y-type electric maze. The results showed that acupuncture at the “Fengfu” acupoint could significantly improve the memory impairment of dementia-like mice, and significantly increase the low contents of 5-HT, NA, and DA in brain tissue (*P* < 0.05). It is suggested that the mechanism of acupuncture at “Fengfu” acupoint in the treatment of senile dementia may be to increase the content of monoamine neurotransmitters in brain tissue (Von Linstow et al., 2017; Wang et al., 2018; Babić Leko et al., 2021). Zhao et al. (1999) the aged SD rats were stochastically divided into the aged group and the aged acupuncture group, the young SD rats were the young control group, and the elderly acupuncture group was treated with acupuncture at “Yongquan” acupoints of both hindlimbs alternately every other day for 30 days (Esteban et al., 2017). The results prove that the level of monoamine neurotransmitters in the brain of aged acupuncture rats and aged rats were significantly lower than that of young rats (*P* < 0.05). The content of monoamine neurotransmitters in the brain of aged acupuncture rats was significantly higher than that of aged rats (*P* < 0.05). It shows that the excitability of central nervous system activity which is closely related to learning and memory ability decreases after aging, and acupuncture can reduce the extent of its decline (Nazarali and Reynolds, 1992; Storga et al., 1996; Trabace et al., 2007; Dekker et al., 2015; Gruden et al., 2016; Vermeiren et al., 2016).

### Mechanisms of inflammatory action in the brain of patients with AD and acupuncture inhibition of inflammatory responses in brain tissue

**FIGURE 2:**
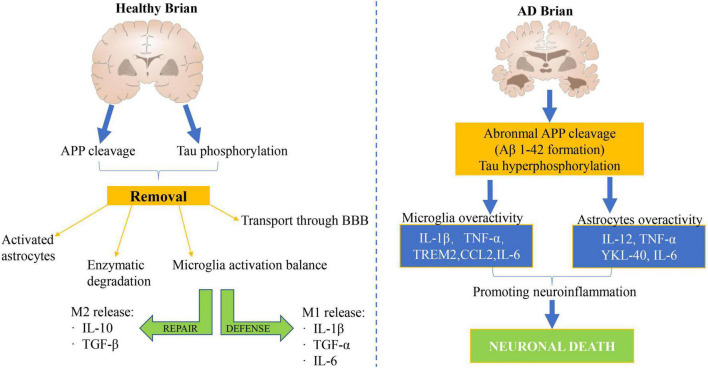
Mechanism of cerebral nerve inflammation in healthy people and patients with AD. A large number of genetic and immunological analyses have demonstrated a significant link between inflammation and AD pathology.

A large number of research have demonstrated that the inflammatory mechanism acts a vital role in the pathogenesis of AD (Cheng et al., 2022; Sun et al., 2022). The neuroinflammatory mechanism of AD has been studied for more than 20 years, but it is still not fully understood. A large number of genetic and immunological analyses show that there is a significant link between inflammation and AD pathology, as shown in Figure 2 (Wang et al., 2022). Modern medical studies have shown that the core pathological mechanism of AD is that Aβ deposition activates microglia to cause inflammation in neuroinflammatory plaques. In the early stage of inflammation, microglia express phagocytic receptors to clear Aβ (An et al., 2022). With the increase of Aβ, Aβ blocks phagocytosis receptors and makes microglia lose phagocytosis. On the contrary, microglia are activated to release inflammatory cytokines, such as IL-1β, IL-6, TNF-α, and so on. These inflammatory factors in turn activate microglia and astrocytes to produce APP and Aβ, forming a malignant positive feedback loop, resulting in a sharp increase in the amount of APP and Aβ (Yousaf et al., 2022). Recent studies have shown that cytokines IL-1 and IL-6 are not only important immune regulatory factors but also have a wide range of central regulatory effects. IL-1 is a kind of cytokine with an immunomodulatory function.

In patients with AD, IL-1 is overexpressed in the receptive area of the cerebral cortex, and the concentration in the tissue increases accordingly (Yue et al., 2022). Tang et al. (2005) used the method of chemical damage to establish the AD rat model, and selected acupoints: “Dazhui,” “Shenshu,” “Taixi,” and “Housanli” acupoints (Wang et al., 2020c). A total of 30 days was a course of treatment. The level of IL-1 and IL-6 in rat brain tissue were tested by radioimmunoassay. The experimental results indicated that the levels of IL-1 and IL-6 in the sham operation group were significantly lower than those in the model group (*P* < 0.01), while the content of IL-1 and IL-6 in the acupuncture group and Hydergine group were significantly lower than those in the model group (*P* < 0.05), but significantly higher than those in the sham operation group (*P* < 0.05) (Cai et al., 2019). The results suggest that the increase of IL-1 and IL-6 levels in brain tissue is closely related to the pathogenesis of AD, which is consistent with the conclusions of previous studies. And acupuncture can significantly reduce the levels of IL-1 and IL-6 in the brain tissue of AD model rats, its effect is similar to that of Hydergine (Jiang et al., 2021; Liao et al., 2022). It is suggested that acupuncture can reduce the levels of IL-1 and IL-6 in the brain tissue of AD rats, which may be one of the effective mechanisms of acupuncture in the treatment of AD. Huang et al. (1998) observe the effect of acupuncture on the inflammatory reaction of AD. RT-PCR (reverse transcriptase polymerase chain reaction) was used to detect the expression of IL-1β and IL-6 in the brain of AD model rats induced by bacterial lipopolysaccharide (Li Y. et al., 2020; Wang et al., 2020b,c; Xie et al., 2021). Acupuncture can reduce the expression of IL-1β and IL-6, suggesting that acupuncture can play a therapeutic role by inhibiting the specific inflammatory reaction in the brain of AD (Fang et al., 2013; He et al., 2017; Cai et al., 2019; Wang et al., 2020a; Yang and Dong, 2020).

### Acupuncture ameliorates neuronal antioxidant damage as well as the free radical scavenging effect

**FIGURE 3:**
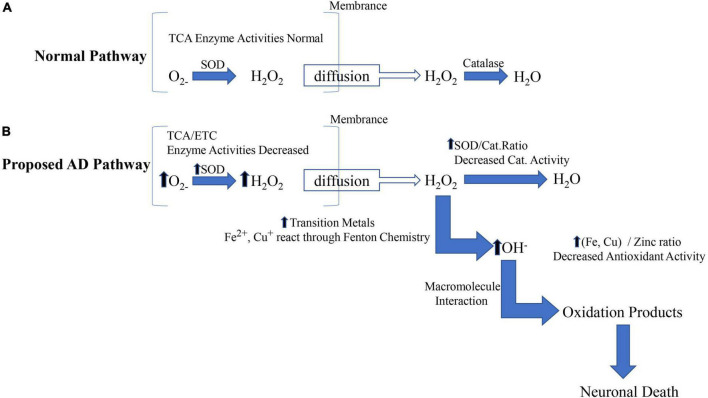
The source and mechanism of cytoplasmic oxidative damage are involved in AD. The mitochondrial (intrinsic) apoptotic pathway includes the release of proapoptotic factors located in the mitochondrial intermembrane space *via* the mitochondrial permeability transition (MPT). Once in the cytoplasm, mitochondrial proteins such as cytC, Smac/Diablo, and Omi/HtrA2 mediate caspase-dependent, whereas endog and AIF induce caspase-independent apoptosis.

Many pieces of evidence suggest that mitochondrial abnormalities are closely related to the disease. However, the significant increase in oxidative damage of neurons is limited to the cytoplasm of susceptible neurons (Aborode et al., 2022; Beura et al., 2022; Estévez-Silva et al., 2022; Maina et al., 2022). We believe that abnormal mitochondria in susceptible neurons play a source role by providing diffusible hydrogen peroxide on the membrane to the surrounding cytoplasm, as shown in Figure 3. The cytoplasm is more vulnerable to hydrogen peroxide because: (1) The cytoplasm is less protected than mitochondria (Kaur et al., 2022); (2) the catalase / SOD ratio decreased in patients with AD, thus reducing the ability to effectively scavenge hydrogen peroxide (Mesa-Herrera et al., 2022; Yoshida et al., 2022); (3) abundant oxidizing metal ions catalyze the Fenton reaction to produce highly active hydroxyl radicals. Therefore, by releasing excess hydrogen peroxide, abnormal mitochondria transmit a series of events involving rich metal ions and cause damage in the cytoplasm (Abu-Elfotuh et al., 2022).

**FIGURE 4:**
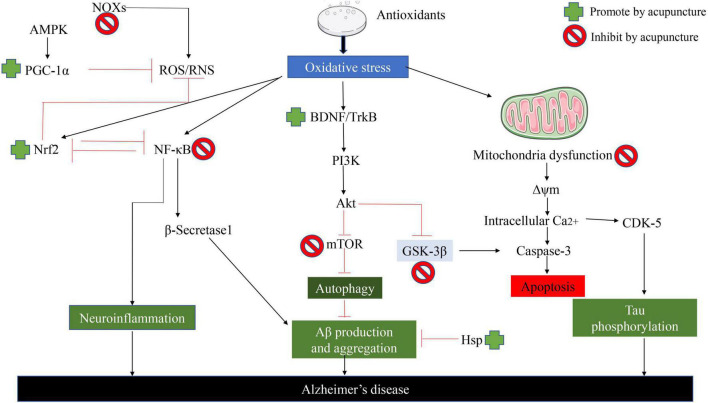
Acupuncture ameliorates AD by suppressing the effects of oxidative stress. Acupuncture can reduce oxidative stress and reduce neuroinflammation through multiple signal transduction pathways, Aβ Production, aggregation, and phosphorylation of Tau.

While the current study showed that acupuncture largely improved the symptoms of AD by inhibiting oxidative stress in AD (Wu et al., 2017). As shown in Figure 4, it is clear to us that acupuncture specifically ameliorates oxidative stress in AD by which pathways and ways: (1) It further alleviates oxidative stress by increasing the synthesis of antioxidant components in the body, thereby reducing the generation of ROS. (2) Acupuncture exerts its effects against oxidative stress by regulating ROS-related signaling pathways and the generation of downstream proteins, thereby reducing apoptosis. (3) Acupuncture directly affects Aβ Protein generation and packing. (4) Acupuncture repairs proteins, lipids, and DNA that are directly damaged by ROS. (5) Acupuncture similarly inhibits oxidative stress by ameliorating neuroinflammation (Tain and Hsu, 2017). In addition to the specific action pathways of acupuncture described above (Nrf2/ARE related signaling pathway), acupuncture can reduce oxidative stress and reduce neuroinflammation through multiple signal transduction pathways, Aβ Production, aggregation, and phosphorylation of tau. Acupuncture can activate Nrf2 and PGC-1α, Inhibit NOx, thereby reducing ROS production. In addition, acupuncture can reverse mitochondrial dysfunction and further decrease the phosphorylation of Tau (Chang et al., 2019).

Oxidative stress acts an increasingly crucial role in the pathogenesis of AD. Lipid peroxidation is not only the inducement of cell membrane aging but also the result of cell aging (Wei et al., 2022; Xin et al., 2022). Lipid peroxidation is induced by oxygen free radicals, and the cytotoxicity of lipid peroxidation products such as MDA plays a vital role in neuronal degeneration and necrosis, leading to the occurrence and development of AD. Wang et al. (2004) used free radical theory and cholinergic theory, to explore the effects of acupuncture on antioxidation and cholinergic system function of the hippocampus and cerebral cortex of pseudo-AD rats (Chang et al., 2019). It was found that the level of MDA in the cerebral cortex of the acupuncture group was significantly lower than that of the model group (*P* < 0.01), while the activity of SOD was significantly increased (*P* < 0.01). In the determination of AchE, the activity of AchE in the model group was significantly lower than that in the acupuncture group (*P* < 0.01) (Yang J. et al., 2019; Zhang et al., 2019). The experimental results show that acupuncture treatment of AD may be achieved through antioxidation. Liu and Fu (2014) made an AD rat model by intraperitoneal injection of D-galactose. The effects of acupuncture at “Baihui,” “Fengfu,” “Shenshu,” and “Xuanzhong” acupoints on the behavior and the contents of serum MDA and T-AOC in AD rats were observed. It turned out that the acupuncture group and acupuncture combined with the medicine group could prolong the latency of the step-down test, reduce the number of errors (*P* < 0.05), decrease the level of serum MDA and increase the content of T-AOC (*P* < 0.05) (Wu et al., 2017). To summarize the above results, acupuncture can improve the level of serum T-AOC, enhance the ability of antioxidation, and reduce the damage of free radicals in AD rats. Guan et al. (2001) in the observation of the effects of acupuncture at “Baihui,” “Dazhui,” and “Mingmen” acupoints on the level of nitric oxide (NO), MDA, and the activity of SOD in the brain tissue of subacute aging mice induced by D-galactose, the experiments proved that the level of NO and MDA in the brain tissue of subacute aging mice increased significantly, while the activity of SOD decreased significantly, which could reverse the above indexes after acupuncture, suggesting that acupuncture at “Baihui,” “Dazhui,” and “Mingmen” acupoints has the effect of anti-brain aging (Ye et al., 2017). Its mechanism may be related to its ability to inhibit the free radical reaction and increase the activity of antioxidant enzymes.

### Inhibition of neuronal apoptosis

**FIGURE 5:**
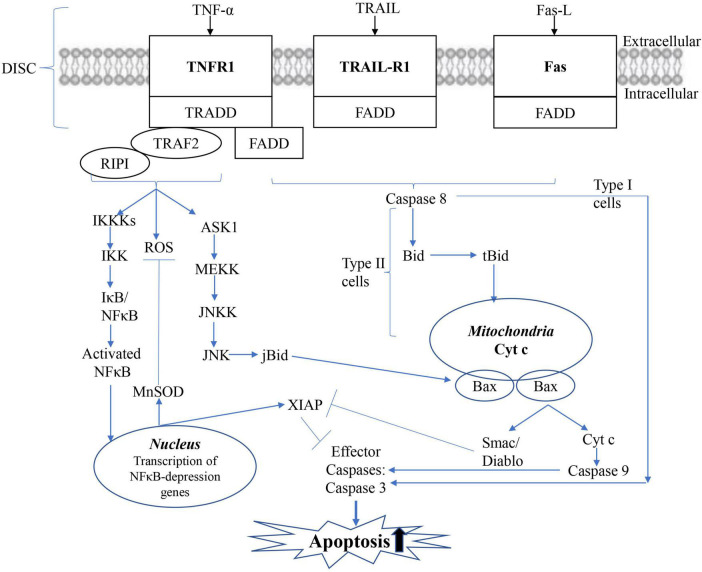
Mitochondrial apoptotic pathway. The mitochondrial (intrinsic) apoptotic pathway includes the release of proapoptotic factors located in the mitochondrial intermembrane space *via* the mitochondrial permeability transition (MPT). Once in the cytoplasm, mitochondrial proteins such as cytC, Smac/Diablo, and Omi/HtrA2 mediate caspase-dependent, whereas endog and AIF induce caspase-independent apoptosis.

Long-term experimental results proved that mitochondria are the critical factors in the early induction and regulation of apoptosis (Li Y. et al., 2022; Weber Boutros et al., 2022). Apoptotic stimuli such as DNA damage, ROS, or Fas signals mediate the death of mitochondrial cells by causing the release of small pro-apoptotic proteins generally located in the intermembrane space of the mitochondria, as shown in Figure 5 (Xiong et al., 2022). Once in the cytoplasm, pro-apoptotic proteins such as Cytochrome c (Cyt c), mitochondrial-derived second caspase activator/low-PI direct IAP binding protein (Smac/Diablo), AIF, and endonuclease G trigger caspase-dependent or independent apoptotic death pathways. In the caspase-dependent mechanism, Cyt c binds to a junction molecule, apoptotic protein activator-1 (APAF-1), to form an apoptotic body. In the presence of ATP or dATP, Caspase 9 is recruited and activated. Caspase 9 further cleaves and activates effector Caspase 3 and/or 7, which treats substrates such as caspase-activated DNA enzyme (ICAD) or PARP, and leads to DNA fragmentation. AIF translocates to the nucleus in a caspase-independent manner, where it induces DNA fragmentation and chromatin agglutination, while ending induces internucleosomal DNA fragmentation.

**FIGURE 6:**
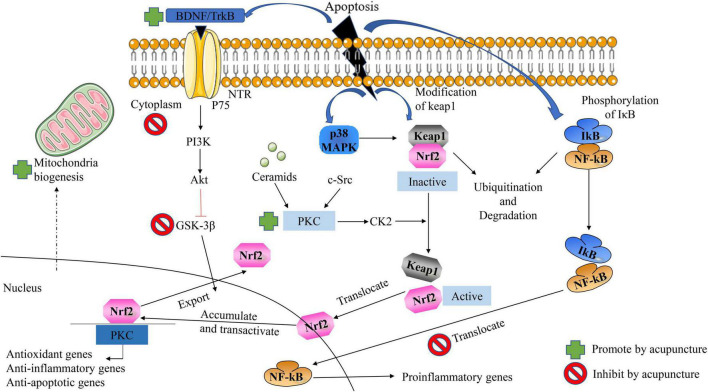
Acupuncture ameliorates neuronal apoptosis in AD by regulating the Nrf2/ARE-related pathway *in vivo*. NFκB the antioxidant effect of Nrf2 can be inhibited by blocking the area region and thereby preventing gene transcription.

It is well known that acupuncture can improve the symptoms of AD patients by inhibiting the apoptosis of nerve cells in a variety of ways and pathways, as shown in Figure 6. (1) On the one hand, increasing the synthesis of antioxidants, and on the other hand, reducing the generation of oxidative stress products, thereby exerting a protective effect on nerve cells. (2) Exerts anti-apoptotic effects on nerve cells by regulating ROS-related signaling pathways and the expression of downstream proteins. (3) Acupuncture inhibition α-Synuclein production as well as accelerating its clearance. The effect of acupuncture on improving neuronal apoptosis in AD *via* the Nrf2/ARE-related pathway is shown in Figure 6 (Ebrahimi-Fakhari et al., 2016). On the other hand, we can also clearly see that acupuncture inhibits GSK-3β by upregulating the expression of BDNF, PI3K/Akt, and Protein Kinase C pathway, further increasing the nuclear translocation, accumulation, and transactivation of Nrf2. In addition, acupuncture can also exert a protective effect on nerve cells by affecting the nuclear translocation of NFkB and thereby downregulating the expression of proinflammatory genes. In addition, acupuncture also promotes mitochondrial biogenesis. Interestingly, the effect of acupuncture on the p38 MAPK pathway has a duality (Gao et al., 2021).

Apoptosis refers to the orderly, cell-autonomous death of cells controlled by genes that maintain homeostasis (Ynag et al., 2021). The apoptosis of brain neurons in patients with AD is 30–50 times higher than that in normal subjects, resulting in a decrease in neurons in the hippocampus, basal forebrain, and neocortex, so apoptosis is one of the important reasons for the decrease in the number of neurons in AD brain tissue (Song et al., 2020; Zhang et al., 2020). It has been found that the changes in morphological structure and function of mitochondria are the key links leading to the disturbance of energy metabolism in the brain, and the disturbance of energy metabolism is closely related to the loss of AD neurons, the formation of senile plaque, nerve fiber tangles and so on. It is well known that apoptosis refers to the programmed death of cells, and as the key molecule “Bcl” of the apoptosis family, one, in turn, divides it into two categories, Bcl2 and Bax, according to the role it plays. Dong et al., found that acupuncture at the “Baihui” and “Shenting” acupoints could directly inhibit apoptosis of cells and thus ameliorate symptoms (improve memory impairment) in MCAO rats by regulating the interaction of Bax and BCL2. Huang et al. (1998) found that by acupuncture model rats (Aβ-140 stimuli) of “Baihui,” “Dazhui,” and “Zusanli” acupoints, which can significantly downregulate Bax protein expression in the rat hippocampus, while upregulating Bcl2 protein expression, thereby inhibiting hippocampal neuronal apoptosis (Yang et al., 2021). Zhang et al. (2017) found that acupuncture at “Baihui,” “Fengfu,” and “Renyu” acupoints significantly decreased the expression levels of Caspase 3 and Bax proteins in the hippocampus of model mice (APP / PS1), which exert protective effects on nerve cells, further improve the symptoms of mice (improved learning and memory ability). In addition to this, numerous studies directly point out that acupuncture exerts anti-apoptotic effects by inhibiting C-Jun amino-terminal protein kinase signaling pathway and thereby inhibiting hippocampal neuronal apoptosis in AD model mice (Huang et al., 2019).

### Activation of hippocampal protein kinase

**FIGURE 7:**
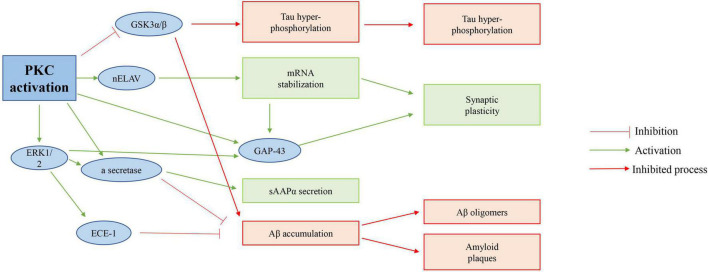
Possible role of PKC activation in AD. Activation of PKC directly inhibits GSK 3β of activity, increases APP protein processing, and in turn inhibits Tau hyperphosphorylation; And reduces Aβ accumulation of proteins.

Protein kinase (PKC) takes part in physiological processes related to cognitive ability (Yang S. et al., 2022). To some degree, PKC is included in so-called cognitive kinases (Lu et al., 2022). It regulates synaptic transmission, and several of its substrates, including Marcks, GAP-43, and NMDA receptors, take part in information processing and storage (Wu et al., 2022). For GAP-43 at least, PKC phosphorylation sites act a crucial role in regulating memory-related tasks, as shown in Figure 7 (Yamahashi et al., 2022).

In recent years, it has been found that the dysfunction of the signal pathway in the brain of senile dementia is closely related to the decrease in the activity of protein kinase. the imbalance between the activity of protein kinase and phosphatase will lead to the hyperphosphorylation of the tau protein, which aggravates the pathological changes of senile dementia (Ortiz-Sanz et al., 2022). Traditional Chinese medicine and acupuncture have certain advantages in the treatment of senile dementia, and whether their mechanism is related to the activation of the suppressed protein kinase signal pathway in the hippocampus is worthy of in-depth study (Zou et al., 2022). Tang et al. (2005) cut off the fimbria-fornix to establish the model of senile dementia. “Baihui,” “Yongquan,” “Taixi,” and “Xuehai” acupoints were selected in the acupuncture group. After 20 times of treatments, the contents of membrane PKC and tyrosine-protein kinase (PTK) in hippocampal tissue cells of each group were measured by radioimmunoassay (Wang et al., 2021). The results show that acupuncture may activate membrane receptors to activate protein kinases such as PKC through corresponding signal transduction pathways and participate in cell growth factor signal transduction: One may be by activating the intracellular PTK connected to it, which can take the receptor itself as the substrate and transduce the signal of tyrosine phosphorylation by PTK, which converts the growth factor signal into an intracellular signal; and maintains the normal metabolism, proliferation, and growth of cells; promote the regeneration of neurons (Li G. et al., 2019; Zheng et al., 2020). The second is that acupuncture promotes the synthesis of nerve growth factor (NGF) in the hippocampus of senile dementia rats. Together with activated PTK, activating PKC; PKC through PLC or IP3-Ca^2+^ pathway can further promote protein synthesis or related gene expression necessary for the formation of learning and memory, to improve the learning and memory impairment of senile dementia (Lin et al., 2015; Zhang et al., 2017).

### Inhibition of microtubule-associated protein expression

**FIGURE 8:**
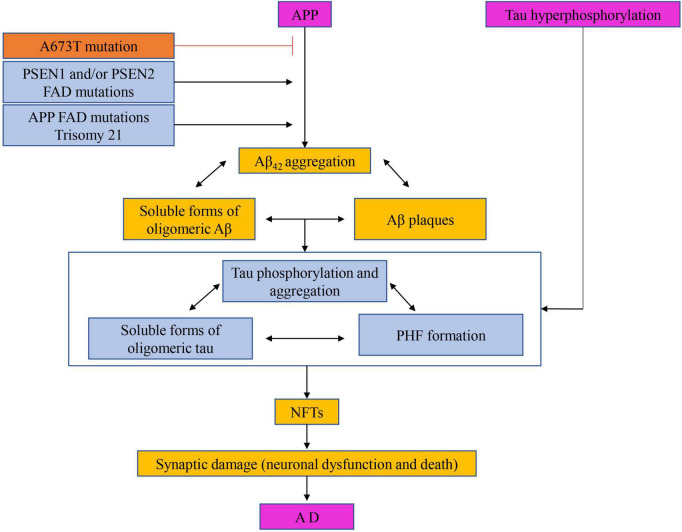
The hypothesis of amyloid cascade with the participation of tau protein. Targeting amyloid precursor secretase thereby prevents or reduces Aβ formation, clearance of amyloid deposits from the brain by active or passive immunization, and prevention or reduction of Aβ aggregation and enhancement of Aβ of clearance.

The Aβ cascade hypothesis suggests that the treatment of AD can start with the restoration of the Aβ balance in the brain (Li Q. et al., 2019; Wang et al., 2021). Based on this, there are mainly four ways to reduce the formation of Aβ, that is, to prevent or reduce the formation of Aβ by targeting amyloid precursor secretase, to clear the deposition of amyloid in the brain by active or passive immunity, to prevent or reduce the accumulation of Aβ and to enhance the clearance of Aβ, as shown in Figure 8 (Zhang et al., 2017).

Current research has demonstrated that the phosphorylation of tau protein is closely related to NFT and AD, and tau protein is the only essential component of NFT (Li R. et al., 2022; Yang B. et al., 2022). Hyperphosphorylation of Tau protein not only reduces its activity of promoting microtubule assembly but also further destroys microtubules by consuming normal tau protein, microtubule-associated protein MAP1, and MAP2, resulting in axoplasmic transport disorder, neuronal process breakage, neuronal degeneration and disintegration, neuronal degeneration and formation of NFT (Teja et al., 2021). Neuronal fiber degeneration caused by NFT act an essential role in the pathogenesis of AD and is parallel to the clinical dementia symptoms of AD patients (Wang et al., 2021). Jiang (2020) taking the AD model rats induced by intraperitoneal injection of D-galactose and intragastric feeding of AlCl_3_ as the research object, acupuncture at “Baihui,” “Dazhui,” “Shenshu,” “Zusanli,” and “Taixi” acupoints (Yu et al., 2020a). The number of Tau protein-positive cells in the hippocampal CA1 region was detected by immunohistochemistry and *in situ* hybridization. The results showed that the expression of Tau protein in the hippocampal CA1 region in the acupuncture group and western medicine group was significantly lower than that in the blank group and model control group (*P* < 0.01). It is suggested that acupuncture can prevent and treat AD by inhibiting the expression of the microtubule-associated protein Tau (Lee et al., 2009; Liu and Fu, 2014; Zhang et al., 2017; Huang et al., 2020; Ma et al., 2020; Yang et al., 2020; Yu et al., 2020b,^2021^).

### Thinking and prospect

As one of the characteristic parts of nonpharmacological therapies and traditional Chinese medicine (TCM), acupuncture plays an effective defensive and therapeutic role for ad with many advantages: (1) It can obviously improve AD symptoms, green and safe with no toxic side effects; (2) Most existing studies of acupuncture have confirmed that acupuncture treatment of ad is not achieved by only one mechanism of action, but by multi-target multi-pathway intervention; (3) All derived from traditional Chinese medicine ideas, according to the characteristics of different etiological disease mechanisms combined with traditional Chinese medicine theory and acupuncture theory as a support based on acupoint selection group formula, is a new idea and method of delaying the progress of AD, and is a new way to prevent and treat AD; (4) Forming a standardized diagnosis and treatment protocol, and making clear the selection of points, operations, parameters, courses, etc., are significant for the promotion of clinical practical applications, it also needs to be continually verified and continually improved in clinical practice, aiming to enrich the means of acupuncture treatment and improve clinical efficacy.

With the development trend of social aging, AD is an urgent problem to be solved in the medical field all over the world. at present, there is no specific drug to treat this disease. Acupuncture with its unique ideas and treatment methods, through a large number of experiments, to verify the reliability of acupuncture treatment of AD. Many advances have been made in the study of the neurobiological mechanism of acupuncture in the treatment of AD, which further proves the good efficacy and unique advantages of acupuncture in the treatment of AD. Animal experiments on related indicators have been widely carried out, but there are few studies on clinical cases, which is also related to the fact that patients and their families do not pay enough attention to the disease. Strengthen the clinical and mechanism research of acupuncture and moxibustion treatment of AD to meet the needs of social development. It is believed that with the progress of research, acupuncture will make new achievements in the treatment of AD.

## Author contributions

All authors listed have made a substantial, direct, and intellectual contribution to the work, and approved it for publication.
